# Real-time identification of life-threatening necrotizing soft-tissue infections using indocyanine green fluorescence imaging

**DOI:** 10.1117/1.JBO.29.6.066003

**Published:** 2024-05-14

**Authors:** Gabrielle S. Ray, Samuel S. Streeter, Logan M. Bateman, Jonathan Thomas Elliott, Eric R. Henderson, NEFARIOUS Study Group

**Affiliations:** a Dartmouth Health, Department of Orthopaedics, Lebanon, New Hampshire, United States; b Dartmouth College, Geisel School of Medicine, Hanover, New Hampshire, United States; c Dartmouth College, Thayer School of Engineering, Hanover, New Hampshire, United States

**Keywords:** fluorescence-guided surgery, dynamic contrast-enhanced fluorescence imaging, indocyanine green imaging, necrotizing soft-tissue infections

## Abstract

**Significance:**

Necrotizing soft-tissue infections (NSTIs) are life-threatening infections with a cumulative case fatality rate of 21%. The initial presentation of an NSTI is non-specific, frequently leading to misdiagnosis and delays in care. No current strategies yield an accurate, real-time diagnosis of an NSTI.

**Aim:**

A first-in-kind, observational, clinical pilot study tested the hypothesis that measurable fluorescence signal voids occur in NSTI-affected tissues following intravenous administration and imaging of perfusion-based indocyanine green (ICG) fluorescence. This hypothesis is based on the established knowledge that NSTI is associated with local microvascular thrombosis.

**Approach:**

Adult patients presenting to the Emergency Department of a tertiary care medical center at high risk for NSTI were prospectively enrolled and imaged with a commercial fluorescence imager. Single-frame fluorescence snapshot and first-pass perfusion kinetic parameters—ingress slope (IS), time-to-peak (TTP) intensity, and maximum fluorescence intensity (IMAX)—were quantified using a dynamic contrast-enhanced fluorescence imaging technique. Clinical variables (comorbidities, blood laboratory values), fluorescence parameters, and fluorescence signal-to-background ratios (SBRs) were compared to final infection diagnosis.

**Results:**

Fourteen patients were enrolled and imaged (six NSTI, six cellulitis, one diabetes mellitus-associated gangrene, and one osteomyelitis). Clinical variables demonstrated no statistically significant differences between NSTI and non-NSTI patient groups (p-value≥0.22). All NSTI cases exhibited prominent fluorescence signal voids in affected tissues, including tissue features not visible to the naked eye. All cellulitis cases exhibited a hyperemic response with increased fluorescence and no distinct signal voids. Median lesion-to-background tissue SBRs based on snapshot, IS, TTP, and IMAX parameter maps ranged from 3.2 to 9.1, 2.2 to 33.8, 1.0 to 7.5, and 1.5 to 12.7, respectively, for the NSTI patient group. All fluorescence parameters except TTP demonstrated statistically significant differences between NSTI and cellulitis patient groups (p-value<0.05).

**Conclusions:**

Real-time, accurate discrimination of NSTIs compared with non-necrotizing infections may be possible with perfusion-based ICG fluorescence imaging.

## 1 Introduction

### 1.1 Necrotizing Soft-Tissue Infections

Necrotizing soft-tissue infections (NSTIs) are aggressive infections with a cumulative case fatality rate near 21%^1^ (range, 6% to 76%).^2^^–^^9^ An NSTI occurs when highly virulent, exotoxin-producing bacteria are introduced to muscle, fascia, or subcutaneous tissue from penetrating wounds or following relatively minor, non-penetrating soft tissue injuries. NSTIs may be polymicrobial (i.e., caused by mixed aerobic/anaerobic pathogens) or monomicrobial (i.e., caused by a single pathogen such as group A *Streptococcus*, clostridial species, and others). Risk factors include blunt trauma, surgical incisions, insect bites, superficial cuts and abrasions, injection sites, muscle strains, pre-existing ulcers or fistulas, peri-rectal abscesses, burns, splinters, chicken pox, childbirth, penetrating injuries, diabetes, and other immunosuppressing conditions.^6^^,^^9^^–^^15^ Disruption of the barrier function provides microbial access to deeper soft tissues, though not all patients have such defined portals of bacterial entry, which makes them at higher risk for delayed diagnosis and treatment. Bacterial proliferation with concomitant exotoxin production leads to rapid disease progression, often within hours—causing widespread destruction of muscle, fascia, and skin; sepsis; multi-organ failure; and death. People of all ages and backgrounds can contract an NSTI, including otherwise healthy individuals, children, and neonates.^8^^,^^10^^,^^11^^,^^15^ The extreme virulence, morbidity, and mortality of NSTIs derive from the ability of causative bacteria to dysregulate microvascular function leading to irreversible tissue perfusion deficits^9^ and to commandeer the body’s acute-phase immune response such that the infection circumvents the body’s typical containment mechanisms against the spread of infection.^16^ The emergent nature and relatively low prevalence of NSTI (0.3 to 15.5 cases per 100,000 people, depending on the geographical location) make studying these infections and establishing advancements in care challenging.^9^

### 1.2 Standard-of-Care for NSTIs

At present, standard-of-care management for NSTIs is twofold: (1) immediate, broad-spectrum intravenous (IV) antibiotics and (2) emergent surgical debridement of affected tissues. Surgical debridement aims to remove all causative bacteria without concern for the preservation of important structures, such as major blood vessels and nerves.^13^ In cases of NSTI affecting an extremity, amputation is required in 19% to 26% of cases.^1^^,^^17^

Multiple factors determine the likelihood of survival with an NSTI; the most critical is rapid diagnosis and emergent initiation of treatment—broad-spectrum IV antibiotics and surgical debridement.^1^^,^^6^ Based on a meta-analysis of 16 NSTI clinical studies, surgical treatment within 12 h of presentation resulted in a statistically significant reduction in mortality (19% versus 34% for delayed surgical treatment).^1^

### 1.3 Challenge of Real-Time Diagnosis: Exams, Imaging, Frozen Section Pathology, and the LRINEC Score

Real-time NSTI diagnosis is challenging and often delayed because patients commonly present with non-specific signs and symptoms (e.g., fever, pain, superficial erythema, and elevated inflammatory laboratory values), especially among patients lacking a defined portal of bacterial entry. Therefore, NSTIs may easily be missed initially or mistaken for other superficial infections, such as cellulitis, which are less emergent and are usually treated with antibiotics alone.^18^ In two series, only 15% to 25% of patients were diagnosed correctly at the time of presentation; 75% to 85% of patients were classified as having a delay in diagnosis.^6^^,^^19^ Delays in management allow bacteria to proliferate and advance centrally, quickly leading to sepsis, irreversible organ failure, and ultimately death. Because of the high stakes of ascertaining a diagnosis of NSTI, clinical investigators have evaluated numerous strategies for NSTI diagnosis, including vital signs, physical examination, radiological imaging, frozen section histology, and clinical scoring systems.^9^^,^^20^^,^^21^

Vital signs and physical examination are non-specific for NSTI, with little diagnostic accuracy (Table S1 in the Supplementary Material).^10^^,^^22^^–^^24^ The presence of bullae, ecchymoses, and hypotension have high specificity for NSTIs; however, these are late indicators of tissue destruction, are associated with high mortality, and are therefore unsuited for early NSTI diagnosis.^10^^,^^23^^,^^25^^,^^26^

Radiographs (i.e., projection X-ray imaging) and computed tomography (CT) are used routinely to evaluate patients with suspected NSTIs. Pooled specificities for radiographs and CT are 91% and 95%, respectively, but pooled sensitivities are marginal for both imaging modalities (62% for radiography and 71% for CT; results based on a meta-analysis of four radiography and seven CT studies; Table S1 in the Supplementary Material).^20^^,^^27^^–^^29^ Magnetic resonance imaging (MRI) provides higher pooled sensitivity (86%) but lower pooled specificity (65%) based on a recent meta-analysis of six studies.^30^ Consensus guidelines for CT-based or MRI-based NSTI diagnosis have not been established to date. In addition, the time requirements for imaging, particularly MRI, lead to delays in care.^31^

Tissue biopsy with frozen section histology was initially reported to be an accurate method of diagnosis,^21^^,^^32^ but more recent clinical study results report it to be less reliable, with low sensitivity (32%).^29^

The Laboratory Risk Indicator for Necrotizing Fasciitis (LRINEC) score is the most highly cited and evaluated tool for identifying NSTIs and is based on laboratory test results (see Table S2 in the Supplementary Material).^22^ However, the LRINEC score yields marginal overall diagnostic performance, particularly sensitivity. A meta-analysis of 23 clinical studies of adult NSTI patients reported pooled sensitivity and specificity of 68% and 85%, respectively, using the standard LRINEC≥6 cutoff. Using the same dataset, if LRINEC≥8 had alternatively been used for diagnosis, pooled sensitivity and specificity were 41% and 95%, respectively.^20^ Furthermore, relatively few studies have prospectively evaluated the scoring system for NSTI identification.^28^^,^^33^^,^^34^

The gold standard for diagnosis of NSTIs requires concordant tissue culture and permanent section histopathology,^29^ both of which require invasive testing (i.e., tissue biopsy and therefore a high degree of suspicion) and several days to provide a result. No current strategies yield an accurate, real-time diagnosis.

### 1.4 Fluorescence Guidance with Indocyanine Green

Fluorescence-guided surgery (FGS) is a rapidly evolving and growing field focused on improving surgical efficacy and safety through enhanced visual recognition of critical structures (e.g., tumors, nerves, blood vessels) or physiological phenomena (e.g., enzymatic activity and vascular/luminal patency). FGS utilizes fluorescent probes (fluorophores) with varied mechanisms for tissue identification, including non-targeted intravascular/intraluminal perfusion probes, molecular-targeted probes, and probes activated by enzymatic or metabolic activity. Today, multiple FGS imaging systems are Food and Drug Administration (FDA)-approved for clinical use or are in trials.^35^^–^^38^

Indocyanine green (ICG) is a non-targeted, intravascular, near-infrared fluorophore with an excellent safety record and >60 years of FDA approval for angiography, ophthalmology, and liver function assessment.^39^ ICG has a peak spectral absorption at ∼800  nm and peak spectral emission at ∼820  nm in blood.^40^ Within seconds of IV administration, ICG enables the visual demarcation of local vasculature, distinguishing perfused tissues from non-perfused tissues. ICG dynamic contrast-enhanced fluorescence imaging (DCE-FI) involves collecting fluorescence image videos for seconds to minutes to measure perfusion first-pass kinetic parameters in target tissues.^41^^–^^43^

ICG fluorescence imaging for the management of NSTI was first reported by Aka et al.^44^ in 2021. They used ICG fluorescence to distinguish between viable and nonviable tissues to guide repeat surgical debridement in a single NSTI case. The present study advances on the initial report by Aka et al.^44^ in the following ways: (1) hypothesizing how NSTI pathophysiology at the histological level impacts perfusion and enables ICG DCE-FI as a potential diagnostic tool; (2) imaging patients with NSTI in a cohort-control design; and (3) analyzing fluorescence imaging data using an established DCE-FI technique.

A distinguishing histological feature of NSTIs is widespread, microvascular thrombosis—occlusion of capillaries and smaller blood vessels within the soft tissues—initiated by the effects of bacterial exotoxins on platelets, leukocytes, and endothelial cells^45^^,^^46^ and further promulgated by NSTI-activated endothelial cell production of tissue factor.^47^ We hypothesized that detectable fluorescence signal voids would occur in NSTI-affected tissues following the administration and imaging of ICG fluorescence. Herein, we report recent results from a clinical pilot study to test this hypothesis.

## 2 Methods

### 2.1 Study Design

**Fig. 1:**
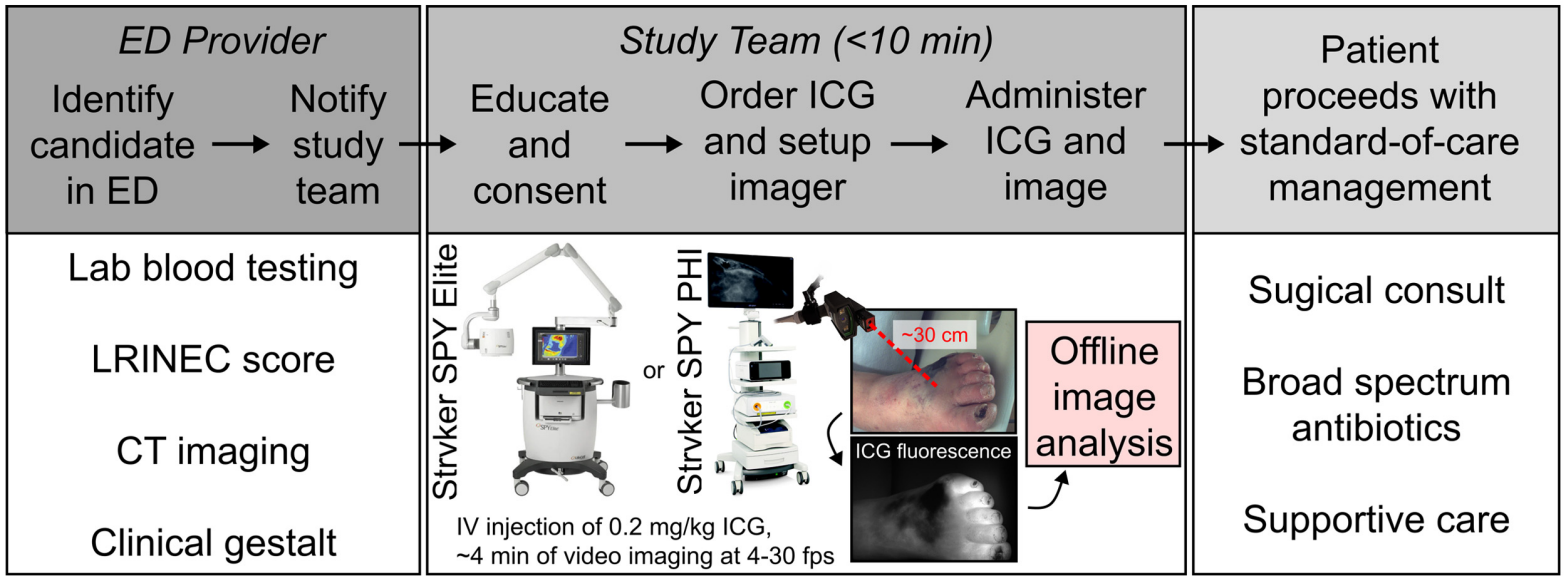
Study overview. Education, consent, enrollment, and imaging of patients suspected of having NSTIs took less than 10 min. ED, Emergency Department; LRINEC, Laboratory Risk Indicator for Necrotizing Fasciitis; CT, computed tomography; ICG, indocyanine green; IV, intravenous injection; FPS, frames per second; DCE-FI, dynamic contrast-enhanced fluorescence imaging.

All aspects of this first-in-kind pilot study adhered to a Dartmouth Health Institutional Review Board-approved protocol. The study (ClinicalTrials.gov identifier: NCT04839302) followed a prospective, non-randomized, observational design to test the hypothesis that measurable fluorescence signal voids occur in NSTI-affected tissues following IV administration and imaging of ICG fluorescence (Fig. 1). Patients presenting to the Emergency Department (ED) at Dartmouth Hitchcock Medical Center, a tertiary care academic hospital in Lebanon, New Hampshire, who were suspected of having an NSTI were identified by ED providers. ED providers notified study team members. Study team members educated and consented eligible patients. Inclusion criteria were as follows: (1) age≥18 years; (2) clinical suspicion of NSTI warranting hospital admission for (a) observation due to suspected NSTI, (b) soft-tissue biopsy, and/or (c) surgical debridement; and (3) ability to give written informed consent (or have legal decision maker present). Exclusion criteria were as follows: (1) history of allergy to ICG and/or iodine^48^ and (2) pregnant women or nursing mothers.

Systemic ICG is cleared by the liver and excreted via bile.^49^ Thus, liver function impacts ICG perfusion kinetics, particularly over longer time lengths (i.e., 10s of minutes or more). Liver failure was not considered an exclusion criterion in this study but was retrospectively documented for all patients enrolled in the study.

Enrolled patients received an IV dose of ICG and were imaged using a portable, cart-based, open-field, FDA-approved imager (a SPY Elite or SPY PHI, Stryker, Kalamazoo, Michigan, United States). The imaged field of view was centered on the region of tissue with visible signs of infection (e.g., inflammation, tissue damage). Initial ICG dosing of 0.5  mg/kg resulted in detector saturation. Dose reduction to 0.2  mg/kg consistently yielded full dynamic range imaging with minimal saturation. Fluorescence imaging at 4 to 30 frames per second occurred for ∼10  s prior to IV administration of ICG and continued for ∼4  min post-administration to measure first-pass perfusion kinetics in the affected and adjacent tissues. A working distance of ∼30  cm was maintained for all cases. In the case of the handheld SPY PHI imager, a three-dimensional (3D)-printed attachment with a laser range finder (see center panel in Fig. 1) was used to maintain the appropriate working distance. DCE-FI analysis was performed “offline” (i.e., not in the clinical theater). The patient engagement did not exceed 10 min per case. After imaging, each patient proceeded with standard-of-care management. The clinical team was blinded to the fluorescence imaging, and imaging did not influence the care delivered to patients.

### 2.2 Dynamic Contrast-Enhanced Fluorescence Imaging

ICG fluorescence image data were processed to create wide field-of-view DCE-FI parameter maps using a custom MATLAB script (v2023a, MathWorks, Natick, Massachusetts, United States).^41^^–^^43^ Specifically, ingress slope (IS), time-to-peak (TTP) intensity, and maximum fluorescence intensity (IMAX) were quantified. In addition, a representative, high-contrast, raw fluorescence image (i.e., a “snapshot”) was manually selected from the video recording as a fourth parameter map for analysis. These steps are illustrated in Figs. S1(a)–1(b) in the Supplementary Material.

### 2.3 Clinical and Image Data Analysis

Clinical variables recorded from each patient included basic demographics (age, sex), the presence of comorbidities (diabetes mellitus, peripheral vascular disease), and standard-of-care laboratory blood test results used to derive the LRINEC score (C-reactive protein level, white blood cell count, hemoglobin level, sodium level, creatinine level, and glucose level; see Table S2 in the Supplementary Material for LRINEC scoring system details).^22^

The ICG fluorescence image data—in the form of the four parameter maps described above (snapshot, IS, TTP, and IMAX)—were further analyzed using custom MATLAB scripts. Tissue pixels were isolated using manually defined regions of interest (ROIs). Using each patient’s representative, high-contrast fluorescence snapshot, ROIs of markedly decreased fluorescence intensity, if present, were delineated by eye and labeled “lesion” ROIs. If no signal voids were present, the tissue region in the center of the field of view was selected as the “lesion” ROI. ROIs of background and lesion tissues were then randomly sampled in pairs to derive signal-to-background ratio (SBR) distributions for all four parameters. These SBR values quantified the contrast to tissues affected by soft tissue infection relative to surrounding tissues within each patient. These steps are illustrated in Fig. S1(c) of the Supplementary Material.

### 2.4 Statistical Analysis

Statistical analysis was implemented in three steps. First, median values for all clinical variables were statistically evaluated between the NSTI patient group and (1) all non-NSTI patients and (2) only cellulitis patients using the Mann–Whitney U test. Second, median parameter map values from lesion ROIs were statistically evaluated between the NSTI patient group and (1) all non-NSTI patients and (2) only cellulitis patients using the Mann–Whitney U test. Third, median lesion ROI values were also used for receiver operating characteristic (ROC) curve analysis to quantify the discriminatory power of each parameter for separating NSTI cases from non-NSTI cases. An optimal ROC curve threshold that balanced sensitivity and specificity was used in all cases unless otherwise stated. All statistical tests were implemented using the Statistics and Machine Learning Toolbox (v12.5) in MATLAB. A p-value<0.05 indicated statistical significance.

## 3 Results

A total of 16 patients were enrolled between August 2021 and August 2023. Two of the 16 patients withdrew from the study before data collection; thus, data from 14 patients were analyzed (average age of 57 years; age range of 34 to 78 years; 57% male; Table 1). Six of the 14 patients had confirmed NSTIs (43%), six had cellulitis (43%), and two had other diagnoses (14%; one case of sterile, diabetes mellitus-associated gangrene and one case of osteomyelitis). Comorbidities and laboratory blood test results for the NSTI, non-NSTI, and cellulitis only patient groups are listed in Table 2; none revealed a statistically significant difference between groups. See Table S3 in the Supplementary Material for enrolled patients’ individual laboratory blood test results used to derive LRINEC scores. See Table S4 in the Supplementary Material for enrolled patients’ liver disease status; no enrolled patients suffered from liver failure, a condition known to impact ICG fluorescence perfusion kinetics.^49^ See Table S5 in the Supplementary Material for standard-of-care imaging performed for each patient as a component of their NSTI workup. See Table S6 in the Supplementary Material for a list of causative pathogens confirmed by tissue biopsy and culture for all NSTI cases.

**Table 1:** Enrolled patient demographics.

Patient number	Sex (female/male)	Age (years)	Diagnosis	Infection location	Comorbidities (yes/no)		LRINEC score
					Diabetes mellitus	Peripheral vascular disease	
01	F	75	Cellulitis	Left lower extremity	Y	N	9
02	F	59	NSTI	Left lower extremity	Y	N	5
03	*Consent withdrawn*						
04	F	78	NSTI	Left lower extremity	N	N	6
05	M	36	Cellulitis	Left lower extremity	Y	N	1^a^
06	M	71	Cellulitis	Left lower extremity	N	Y	6
07	F	62	NSTI	Right lower extremity	Y	Y	8
08	M	48	Gangrene	Left hallux	Y	Y	5
09	M	45	Cellulitis	Left lower extremity	Y	N	8
10	M	34	Cellulitis	Left lower extremity	N	Y	9
11	F	54	Cellulitis	Groin	N	N	2
12	M	61	NSTI	Left foot	Y	N	7
13	F	68	Osteomyelitis	Left foot	Y	Y	8
14	M	53	NSTI	Groin	N	N	2^a^
15	*Consent withdrawn*						
16	M	60	NSTI	Right lower extremity	Y	N	7

a C-reactive protein (CRP) values not available for this patient. Thus, the LRINEC score was calculated without the CRP point contribution.

**Table 2:** Comorbidities and laboratory blood test results for the NSTI, non-NSTI, and cellulitis only patient groups. Statistical test results (p-values) reflect comparisons to the NSTI patient group.

Clinical variable	NSTI group (n=6)	Non-NSTI group (n=8)	p-Value	Cellulitis only patient group (n=6)	p-Value
*Comorbidity, n (%)*					
Diabetes mellitus	4 (67%)	5 (63%)	1.00	3 (50%)	1.00
Peripheral vascular Disease	1 (17%)	4 (50%)	0.48	2 (33%)	0.48
*Laboratory finding, mean ± std. dev.*					
CRP level (mg/L)^a^	213.3 ± 96.1	202.7 ± 79.9	0.83	208.5 ± 56.2	0.69
WBC count (cells/μL)	16.1 ± 7.0	17.7 ± 6.9	0.66	16.1 ± 5.9	0.94
Hemoglobin level (g/dL)	11.5 ± 2.4	13.1 ± 1.7	0.24	13.0 ± 1.9	0.26
Sodium level (mmol/L)	135.2 ± 4.4	132.0 ± 3.2	0.22	132.3 ± 3.7	0.35
Creatinine level (mg/dL)	1.4 ± 0.8	1.3 ± 0.7	0.98	1.4 ± 0.8	0.85
Glucose level (mg/dL)	225.8 ± 225.9	189.8 ± 117.7	0.85	184.7 ± 120.2	0.82
*Clinical score, mean ± std. dev.*					
LRINEC	5.8 ± 2.1	6.0 ± 3.1	0.68	5.8 ± 3.5	0.72

CRP, C-reactive protein; WBC, white blood cell count.

a CRP level not recorded for two patients; see Table 1 above.

**Fig. 2:**
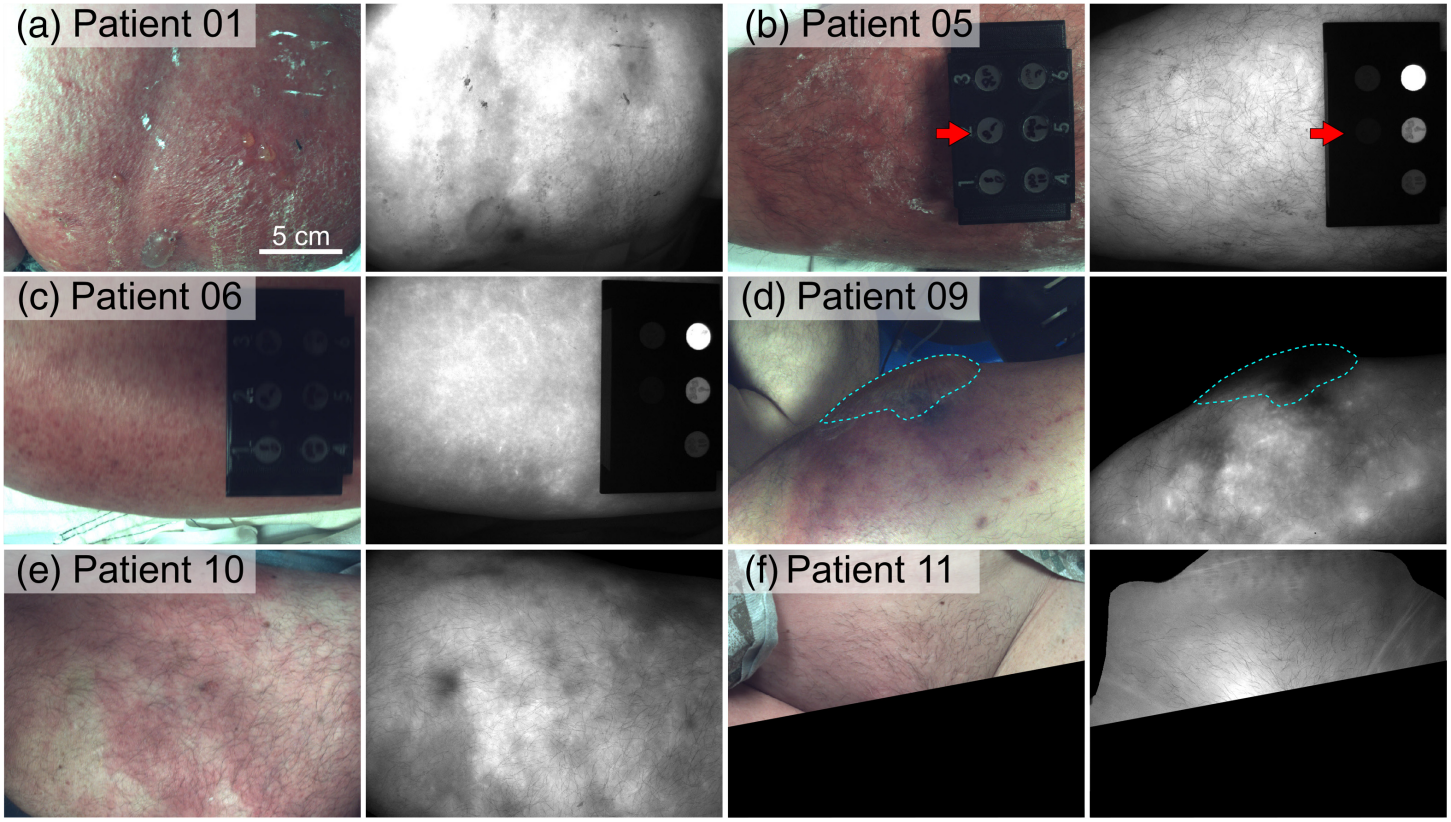
(a)–(f) White light color images and representative ICG fluorescence images (snapshots) of confirmed cases of cellulitis. Red arrows annotate an ICG-equivalent, 3D-printed fluorescence standard, pictured in select cases in the study. (d) Patient 09 had a surface tissue bulla delineated by the cyan dashed line. The scale bar in panel (a) applies to all images.

**Fig. 3:**
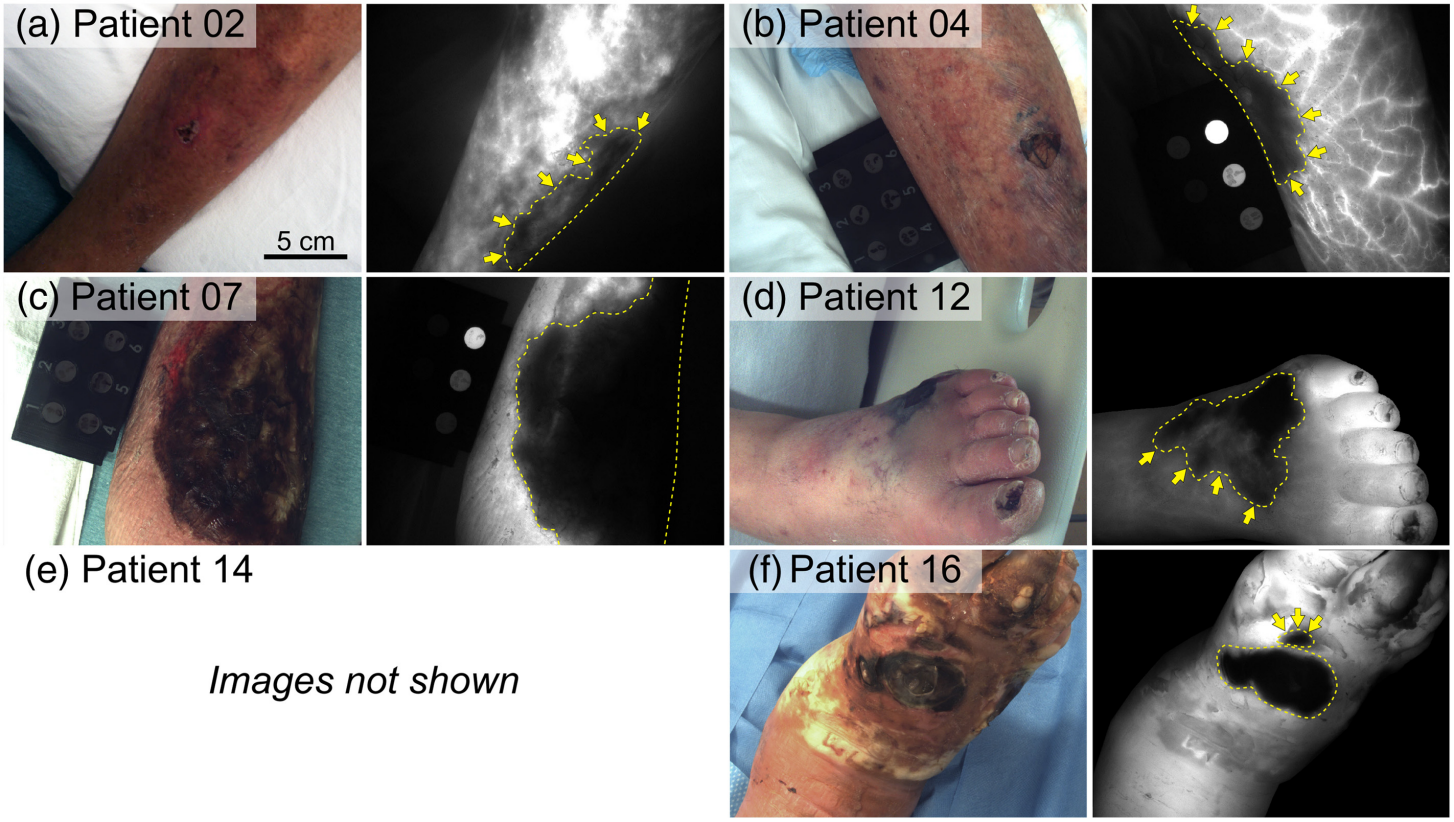
(a)–(f) White light color images and representative ICG fluorescence images (snapshots) of confirmed cases of NSTI. Yellow dashed lines delineate fluorescence signal voids indicative of diminished blood perfusion caused by infection. Yellow arrows highlight affected tissues not clearly resolved in white light color images. (e) Patient 14 images are not shown because the infection involved the genitalia. The scale bar in panel (a) applies to all images.

**Fig. 4:**
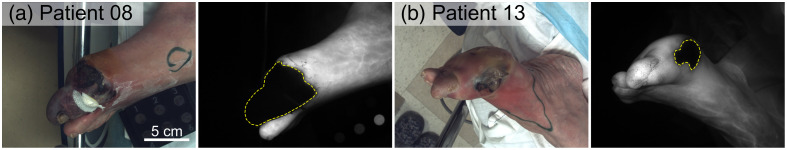
(a)–(b) White light color images and representative ICG fluorescence images (snapshots) of confirmed cases of other tissue infections. (a) Patient 08 is a case of sterile, diabetes mellitus-associated gangrene. (b) Patient 13 is a case of osteomyelitis. The scale bar in panel (a) applies to all images.

White light color images and ICG fluorescence snapshots of confirmed cases of cellulitis, NSTI, and other diagnoses are shown in Figs. 2–4, respectively. All cellulitis cases exhibited increased overall fluorescence intensity without the presence of signal voids in affected tissues, whereas all NSTI cases contained prominent fluorescence signal voids (see yellow lines and arrows in Fig. 3). An ICG-equivalent, 3D-printed fluorescence standard (QUEL Imaging, White River Junction, Vermont, United States) was pictured in some cases [e.g., Fig. 2(b)] but was not available during all imaging sessions. The cellulitis case in Fig. 2(d) had a bulla readily visualized under room light that yielded an ICG fluorescence signal void; this void was not considered a lesion due to clinical context (i.e., blood perfusion is not expected in bullae). The cases with other diagnoses (diabetes mellitus-associated gangrene, osteomyelitis) also exhibited prominent ICG fluorescence signal voids.

**Fig. 5:**
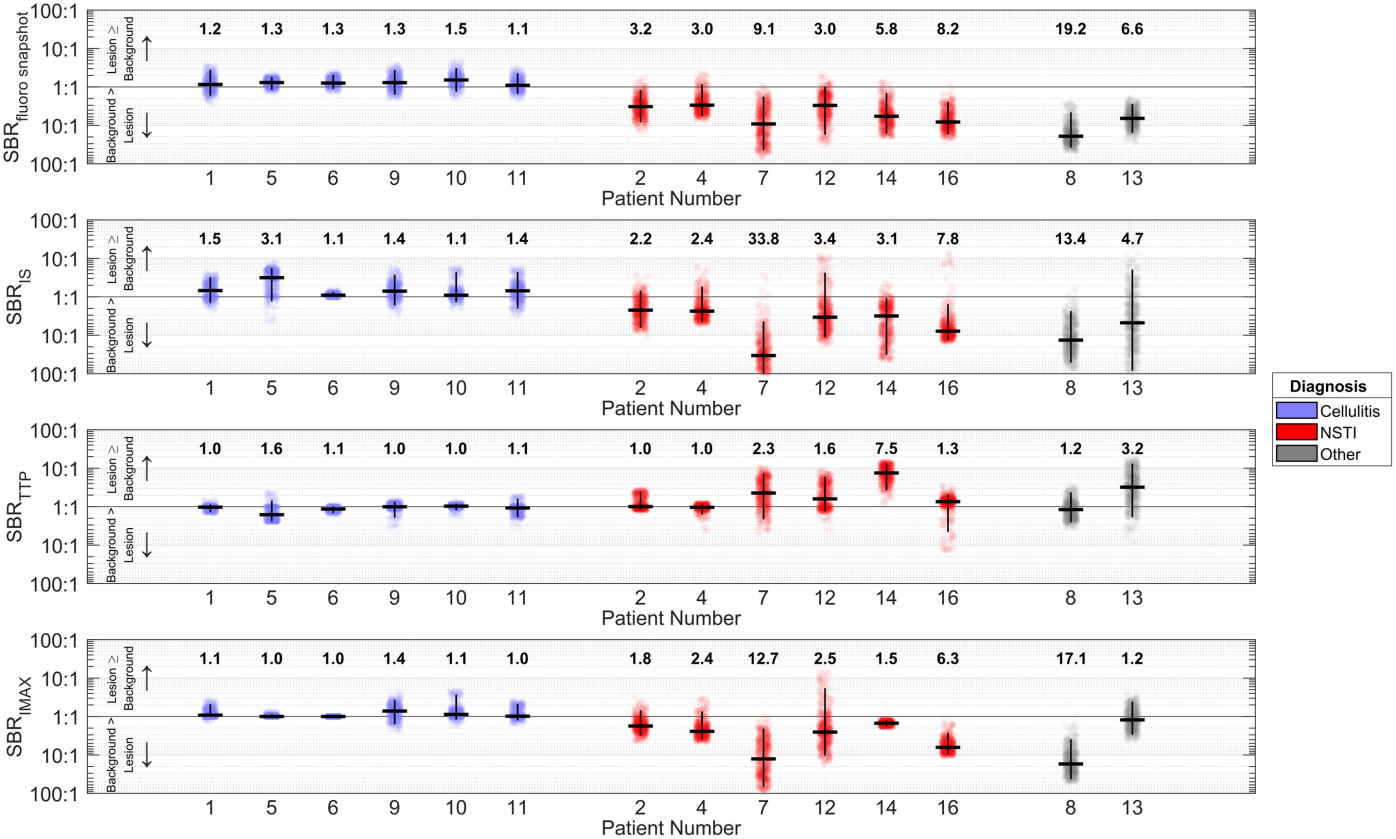
SBR distributions for cases that exhibited prominent ICG fluorescence signal voids, including all NSTI cases, one case of diabetes mellitus-associated gangrene (patient 08), and one case of osteomyelitis (patient 13). SBR distributions were determined by paired random sampling of lesion and background tissue ROIs. The horizontal black lines denote distribution medians; these median SBRs are also typed above each distribution for clarity. The vertical black lines depict 95% confidence intervals.

SBR distributions from paired ROI sampling are summarized in Fig. 5 for all imaged patients. Median lesion-to-background tissue SBRs based on snapshot, IS, TTP, and IMAX parameter maps ranged from 1.1 to 1.5, 1.1 to 3.1, 1.0 to 1.6, and 1.0 to 1.4, respectively, for the cellulitis group. Meanwhile, these ranges were 3.0 to 9.1, 2.2 to 33.8, 1.0 to 7.5, and 1.5 to 12.7, respectively, for the NSTI patient group. See Figs. S2–S4 in the Supplementary Material for all ICG fluorescence parameter maps and ROI selections used to derive SBR values. Fig. S5 in the Supplementary Material shows representative ICG fluorescence intensity time profiles for cases of NSTI, cellulitis, and gangrene; this figure illustrates that measured fluorescence SBRs stabilize within ∼60  s of ICG ingress into tissue vasculature.

Parameter map median pixel values from only lesion ROIs were aggregated from the NSTI, non-NSTI, and cellulitis only patient groups. Pooled results are shown in Table 3 (see Table S7 in the Supplementary Material for individual patient median “lesion” ROI parameter map values). Snapshot, IS, and IMAX parameters exhibited statistical significance between the NSTI and cellulitis patient groups.

**Table 3:** ICG fluorescence parameter values (mean ± std) based on the lesion region of interest median pixel value from each patient. Parameters include snapshot (a.u.), ingress slope (IS, a.u./s), time-to-peak (TTP, s), and max intensity (IMAX, a.u.). Statistical test results (p-values) reflect comparisons to the NSTI patient group.

Parameter map	NSTI group (n=6)	Non-NSTI group (n=8)	p-Value	Cellulitis only patient group (n=6)	p-Value
Snapshot (a.u.)	24.7 ± 11.1	122.2 ± 73.6	0.13	160.0 ± 27.3	<0.01
IS (a.u./s)	4.2 ± 4.0	24.8 ± 15.2	0.04	32.8 ± 4.7	<0.01
TTP (s)	34.5 ± 35.6	19.5 ± 20.8	0.41	11.9 ± 3.5	0.24
IMAX (a.u.)	71.3 ± 68.8	201.8 ± 89.9	0.02	245.7 ± 22.7	<0.01

**Fig. 6:**
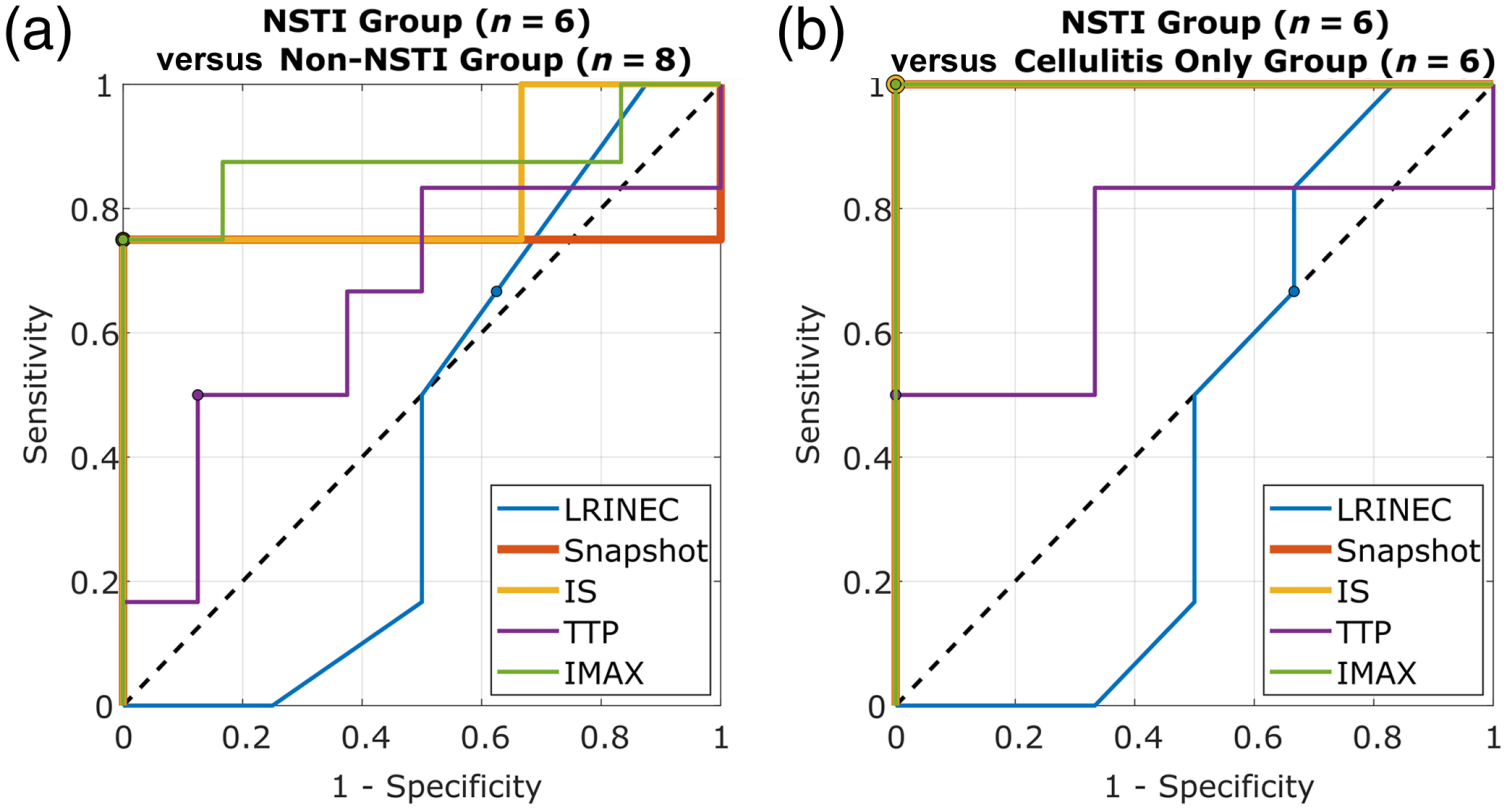
ROC curves for the LRINEC score and ICG fluorescence parameters, based on numbers provided in Table S7 in the Supplementary Material). IS, ingress slope; TTP, time-to-peak; IMAX, maximum intensity. Black dashed lines denote random diagnostic performance. ROC curve optimal cutoff points, denoted by colored circles, were selected to balance sensitivity and specificity, except a standard LRINEC score≥6 cutoff was used.^22^

The ICG fluorescence parameters summarized in Table 3 were directly compared with the LRINEC score for discriminating NSTI-positive versus NSTI-negative patients via ROC curve analysis. ROC curves are shown in Fig. 6; results comparing all NSTI cases versus all non-NSTI cases are shown in Fig. 6(a), and results comparing all NSTI cases versus only cellulitis cases are shown in Fig. 6(b).

Based on the ROC curves shown in Fig. 6, the diagnostic performances of LRINEC scoring and individual ICG fluorescence parameters are summarized in Table 4.

**Table 4:** Diagnostic performance of the LRINEC score and ICG fluorescence parameters (values in Table S7 in the Supplementary Material) based on ROC curve analysis shown in Fig. 5. ROC curve optimal cutoff points were selected to balance sensitivity and specificity, except a standard LRINEC score≥6 cutoff was used.^22^

Parameter map	NSTI group (n=6) versus non-NSTI group (n=8)			NSTI group (n=6) versus cellulitis group (n=6)		
	ROC curve cutoff	Sensitivity (%)	Specificity (%)	ROC curve cutoff	Sensitivity (%)	Specificity (%)
LRINEC score	6 (standard)	67	38	6 (standard)	67	33
Snapshot (a.u.)	132.0	100	75	132.0	100	100
IS (a.u./s)	26.2	100	75	26.2	100	100
TTP (s)	28.8	50	13	28.8	50	0
IMAX (a.u.)	199.3	100	75	199.3	100	100

## 4 Discussion

Results from this pilot study support the hypothesis that detectable fluorescence signal voids occur in NSTI-affected tissues following the administration of ICG and fluorescence imaging. Patient 07 [Fig. 3(c)] had extensive skin necrosis, which would mandate surgical debridement even without imaging. However, in several cases, imaging revealed tissue perfusion deficits associated with NSTI that were not visualized via white light [Figs. 3(a), 3(b), 3(d), 3(f)]. Meanwhile, all cellulitis cases exhibited increased overall ICG fluorescence, indicative of a hyperemic response in affected tissues (Fig. 2).

A fluorescence SBR ≥1.5 is considered useful for distinguishing features in open-field FGS.^50^ As such, all ICG fluorescence snapshot and DCE-FI parameters provide potentially useful contrast that an ED provider or surgeon might use to rapidly identify patients with NSTIs and visualize affected tissues. In the context of NSTI, perfusion-based ICG fluorescence contrast is fundamentally tied to underlying pathophysiology (i.e., microvascular thrombosis). Overall, tissues affected by NSTI exhibited diminished perfusion relative to surrounding tissues and non-necrotizing infections (i.e., cellulitis). This observation was based on lower snapshot and maximum fluorescence intensities and reduced ingress perfusion into affected tissues. Different parameters offered increased SBR depending on the case (Fig. 5), suggesting that combining multiple parameters may improve the detection of NSTI.

The LRINEC score is the most widely evaluated clinical scoring system for detecting NSTIs though its clinical utility in this setting remains controversial. All patients in this study underwent standard-of-care management for suspected NSTI, but not all required lab tests (i.e., C-reactive protein) were conducted to generate complete LRINEC scores for two patients. This finding emphasizes the point that, although the LRINEC score may be a well-studied scoring system, it is not fully implemented clinically. In this study, no statistically significant difference in LRINEC score was observed between NSTI and non-NSTI patient groups (Table 2). If the standard LRINEC score cutoff of ≥6 had been used herein, the sensitivity and specificity for identifying patients with NSTIs (versus all non-NSTI cases) would have been 67% and 38%, respectively [Fig. 6(a), Table 4]. Meanwhile, if median lesion ROI ICG fluorescence snapshot, IS, or IMAX parameter values had been used, the sensitivity and specificity for NSTI identification would have been 100% and 75%, respectively [Fig. 6(a), Table 4]. If NSTI cases were compared with only cellulitis cases, LRINEC≥6 yielded sensitivity and specificity of 67% and 33%, respectively [Fig. 6(b), Table 4]. Meanwhile, fluorescence snapshot, IS, and IMAX parameter values individually provided perfect discrimination between NSTI and cellulitis cases [Fig. 6(b), Table 4].

This study has limitations that warrant discussion. Only patient-level histopathological data were collected in this study per standard of care. Thus, the spatial distribution of histological markers of infection (e.g., bacterial burden, vascular thrombi) is unknown and cannot be co-registered to spatially localized ICG fluorescence parameters. Future work will involve preclinical modeling of NSTI through which these relationships will be determined in a controlled, laboratory setting. This work will also determine whether ICG fluorescence imaging might be useful for determining the spatial extent of NSTI, thereby providing improved guidance during surgical debridement of affected tissues.

ICG perfusion parameters quantified in this study were selected for simplicity and interpretability, but most were defined in terms of arbitrary units (a.u.) of fluorescence. Future work will focus on the quantification of more objective perfusion parameters, such as normalized parameters (e.g., normalized IS)^51^ and/or parameters defined in terms of absolute units (e.g., radiance) using fluorescence imaging standards. An ICG-equivalent, 3D-printed fluorescence standard was imaged in some cases [e.g., Fig. 2(b)] but not all, precluding its use to standardize fluorescence measurements and enable more quantitative inter-patient/measurement comparisons. Motion artifacts precluded the quantification of additional DCE-FI parameters (e.g., the egress slope) for all patients.^41^^,^^42^ In addition, the commercial imagers used in this study provided a limited dynamic range; all images were compressed to eight-bit (0 to 255 range) data. As such, image saturation occurred in some cases. Finally, our data indicated a potential overlap in ICG fluorescence parameters for patients with diabetes mellitus-associated gangrene, severe osteomyelitis with cutaneous involvement, and NSTI. Future work will focus on improving data collection quality and the use of quantitative, ICG-equivalent fluorescence standards to improve inter-patient/measurement comparisons.^52^ Furthermore, we anticipate that achieving maximal diagnostic performance may involve clinical parameters (e.g., vitals, labs, imaging) in combination with fluorescence imaging.

The results presented herein suggest that ICG fluorescence imaging may be effective for real-time identification of NSTI, expanding the physician’s toolkit for addressing the pressing clinical problem of life-threatening NSTI. Our pilot study is limited in terms of case numbers (n=14 images) and diversity (e.g., skin pigmentation, infection location, and depth in tissue). Additional clinical data collection is warranted to evaluate this fluorescence imaging technique on a larger and more diverse patient cohort.

## 5 Conclusion

NSTIs can be life-threatening, rapidly progressing infections for which accurate, real-time diagnosis strategies currently do not exist. ICG fluorescence imaging for surgical guidance is safe for human use and effective for increasing contrast to critical anatomical structures and pathologic phenomena. Real-time, accurate identification of NSTI may be possible with perfusion-based ICG fluorescence imaging.

## Supplementary Material



## Data Availability

Code and raw image data from this study are available from the corresponding author upon reasonable request.
