# Supplementary material for: Real-time identification of life-threatening necrotizing soft-tissue infections using indocyanine green fluorescence imaging
Source: J Biomed Opt. 2024 May 14;29(6):066003. doi: 10.1117/1.JBO.29.6.066003 (PMC11092151; doi:10.1117/1.JBO.29.6.066003)
Supplement: Supplementary file 1 [file JBO_029_066003_SD001.pdf]

Supplemental material for

Real-Time Identification of Life-Threatening Necrotizing Soft-Tissue Infections Using Indocyanine Green Fluorescence Imaging

Gabrielle S. Ray[1,2*], Samuel S. Streeter[1,2*], Logan M. Bateman[3], Jonathan Thomas Elliott[1,2,3], Eric R. Henderson[1,2,3†], and the NEFARIOUS Study Group

1. Department of Orthopaedics, Dartmouth Health, Lebanon, NH 03756, USA
2. Geisel School of Medicine, Dartmouth College, Hanover, NH 03755, USA
3. Thayer School of Engineering, Dartmouth College, Hanover, NH 03755, USA

[*]These authors contributed equally to this work.

[†]Corresponding Author:
Eric R. Henderson, E-mail: Eric.R.Henderson@hitchcock.org

Supplemental Table 1. Performance of various strategies for identifying NSTIs. Results here are compiled primarily from Fernando *et al.*[20] but also from Lam *et al.*,[27] Hsiao *et al.*,[28] and Solomon *et al.*[29]

**Fever**	**Patients**	**Diagnostic Metrics (95% CI)**	
Author		*Sensitivity*	*Specificity*
Wong et al. 2004	314	0.53 (0.42-0.63)	0.58 (0.51-0.64)
Borschitz et al. 2015	88	0.52 (0.33-0.71)	0.71 (0.58-0.82)
Chao et al. 2012	125	0.44 (0.33-0.57)	0.94 (0.84-0.99)
Alayed et al. 2015	120	0.30 (0.17-0.47)	0.76 (0.65-0.85)
**OVERALL**	**647**	**0.46 (0.40-0.53)**	**0.68 (0.63-0.72)**

**Exam (Bullae)**	**Patients**	**Diagnostic Metrics (95% CI)**	
Author		*Sensitivity*	*Specificity*
Chao et al. 2012	125	0.61 (0.49-0.72)	0.70 (0.56-0.82)
McGillicuddy et al. 2011	305	0.27 (0.15-0.43)	0.96 (0.93-0.98)
Wall et al. 2000a	42	0.24 (0.08-0.47)	1.00 (0.84-1.00)
Wall et al. 2000b	359	0.16 (0.05-0.34)	0.97 (0.94-0.99)
Alayed et al. 2015	120	0.10 (0.03-0.24)	0.99 (0.93-1.00)
**OVERALL**	**951**	**0.34 (0.27-0.41)**	**0.95 (0.93-0.96)**

**Exam (Hypotension)**	**Patients**	**Diagnostic Metrics (95% CI)**	
Author		*Sensitivity*	*Specificity*
Cranendonk et al. 2017	54	0.61 (0.42-0.78)	0.70 (0.47-0.87)
Chao et al. 2012	125	0.36 (0.25-0.48)	1.00 (0.93-1.00)
Alayed et al. 2015	120	0.20 (0.09-0.36)	0.98 (0.91-1.00)
Wong et al. 2004	314	0.18 (0.09-0.36)	0.97 (0.94-0.99)
Wall et al. 2000b	359	0.06 (0.00-0.24)	0.99 (0.84-1.00)
Wall et al. 2000a	42	0.05 (0.01-0.21)	1.00 (0.98-1.00)
**OVERALL**	**1014**	**0.25 (0.20-0.31)**	**0.98 (0.96-0.99)**

**Imaging (Radiography)**	**Patients**	**Diagnostic Metrics (95% CI)**	
Author		*Sensitivity*	*Specificity*
Leichtle et al. 2016	108	0.84 (0.73-0.91)	0.34 (0.19-0.52)
Thomas et al. 2012	87	0.35 (0.14-0.62)	1.00 (0.95-1.00)
Wall et al. 2000a	37	0.39 (0.17-0.64)	0.95 (074.-1.00)
Wall et al. 2000b	246	0.32 (0.14-0.55)	0.97 (0.94-0.99)
**OVERALL**	**478**	**0.62 (0.53-0.71)**	**0.91 (0.88-0.94)**

**Imaging (CT scan)**	**Patients**	**Diagnostic Metrics (95% CI)**	
Author		*Sensitivity*	*Specificity*
Carbonetti et al. 2016	36	1.00 (0.69-1.00)	0.96 (0.80-1.00)
Martinez et al. 2017	184	1.00 (0.75-1.00)	0.98 (0.94-0.99)
Zacharias et al. 2010	67	1.00 (0.86-1.00)	0.81 (0.66-0.91)
Wang et al. 2004	22	0.74 (0.49-0.91)	1.00 (0.29-1.00)
Leichtle et al. 2016	86	0.70 (0.59-0.80)	0.42 (0.15-0.72)
Thomas et al. 2012	87	0.65 (0.38-0.86)	0.94 (0.86-0.98)
McGillicuddy et al. 2011	305	0.43 (0.28-0.59)	0.98 (0.96-0.99)
**OVERALL**	**787**	**0.71 (0.65-0.77)**	**0.95 (0.93-0.97)**

**Scoring (LRINEC≥6)**	**Patients**	**Diagnostic Metrics (95% CI)**	
Author		*Sensitivity*	*Specificity*
Carbonetti et al. 2016	36	1.00 (0.69-1.00)	0.54 (0.33-0.73)
Sandner et al. 2015	611	0.94 (0.70-1.00)	0.94 (0.92-0.96)
Wong et al. 2004	140	0.93 (0.83-0.98)	0.92 (0.84-0.97)
Borschitz et al. 2015	88	0.83 (0.64-0.94)	0.90 (0.79-0.96)
Holland et al. 2009	28	0.80 (0.44-0.97)	0.67 (0.41-0.87)
Narashimhan et al. 2017	268	0.76 (0.66-0.85)	0.93 (0.88-0.96)
Zemplenyi et al. 2017	43	0.60 (0.15-0.95)	0.68 (0.51-0.82)
Syed et al. 2017	44	0.59 (0.39-0.78)	0.47 (0.23-0.72)
Liao et al. 2012	1627	0.59 (0.53-0.66)	0.84 (0.82-0.86)
Thomas et al. 2012	19	0.56 (0.21-0.86)	0.60 (0.26-0.88)
Lam et al. 2010	285	0.43 (0.23-0.66)	0.83 (0.78-0.88)
Hsiao et al. 2020	931	0.43 (0.34-0.53)	0.83 (0.80-0.86)
Kim et al. 2013	30	0.43 (0.10-0.82)	0.87 (0.66-0.97)
Neeki et al. 2017	995	0.36 (0.23-0.51)	0.89 (0.87-0.91)
Chao et al. 2012	125	0.17 (0.09-0.27)	1.00 (0.93-1.00)
**OVERALL**	**5270**	**0.60 (0.56-0.64)**	**0.87 (0.86-0.88)**

**Frozen Section Hist.**	**Patients**	**Diagnostic Metrics (95% CI)**	
Author		*Sensitivity*	*Specificity*
Solomon et al. 2018	166	0.32	0.91

**LEGEND**		
**Sensitivity/ specificity**	≥90%	Green
	50-90%	Yellow
	<50%	Red

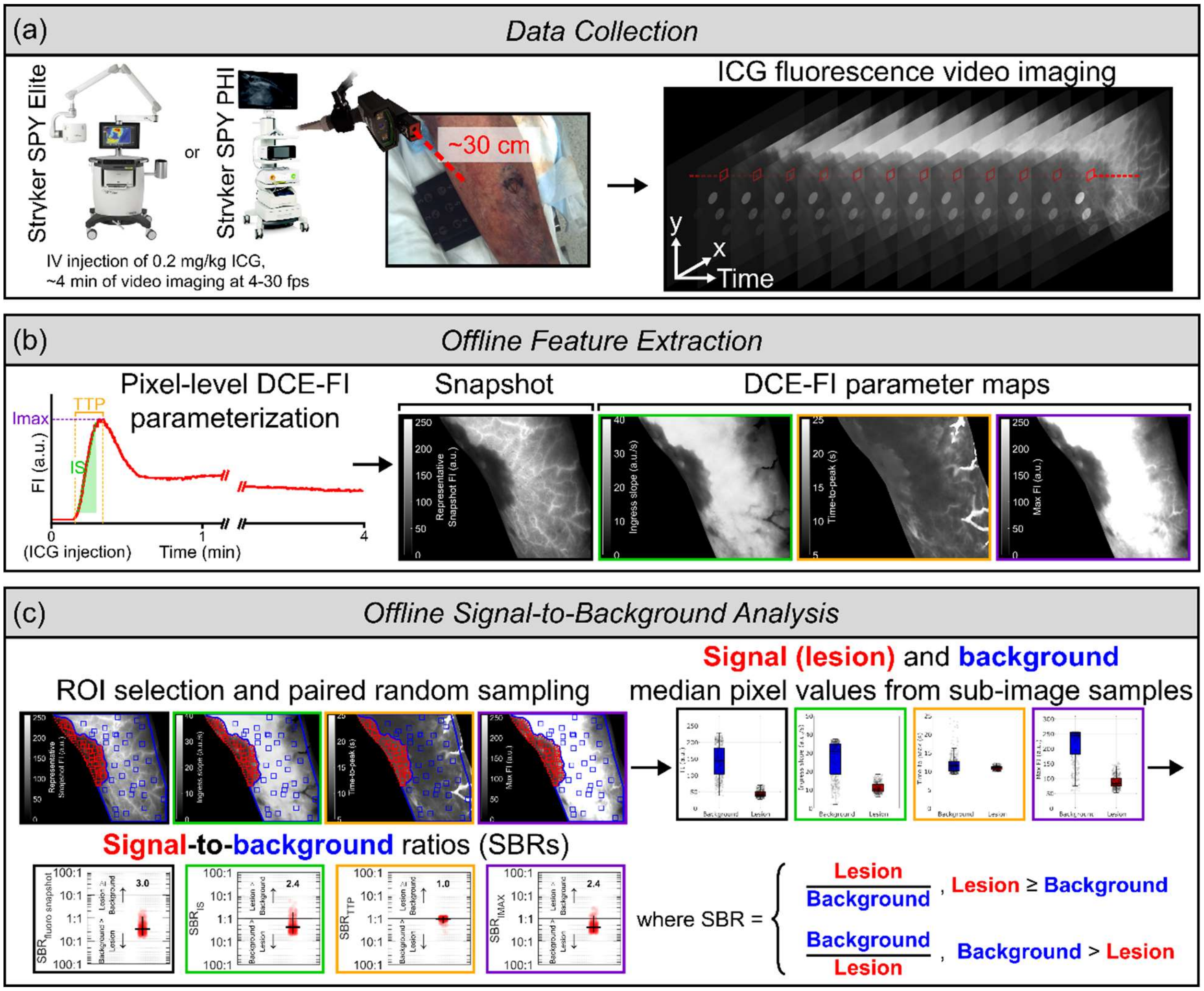

Supplemental Figure 1. Detailed image analysis workflow. Random sampling for signal-to-background ratio (SBR) quantification involved a total of 500 (~1 cm^2) paired samples extracted from the lesion (red) and background tissue (blue) regions of interest (ROIs). IV = intravenous. ICG = indocyanine green. fps = Frames per second. DCE-FI = Dynamic contrast-enhanced fluorescence imaging. FI = Fluorescence intensity. IS = Ingress slope. TTP = Time-to-peak intensity. Imax = Maximum fluorescence intensity. ROI = Region of interest.

Supplemental Table 2. Laboratory Risk Indicator for Necrotizing Fasciitis (LRINEC) score components used for calculating the LRINEC score, as defined by Wong *et al.*[22]

Laboratory Finding	LRINEC Score Contribution (points)
CRP Level (mg/L)	
<150	0
≥150	4
WBC Count (cells/µL)	
<15	0
15-25	1
>25	2
Hemoglobin Level (g/dL)	
>13.5	0
11-13.5	1
<11	2
Sodium Level (mmol/L)	
≥135	0
<135	2
Creatinine Level (mg/dL)	
≤1.6	0
>1.6	2
Glucose Level (mg/dL)	
≤180	0
>180	1

Supplemental Table 3. Laboratory Risk Indicator for Necrotizing Fasciitis (LRINEC) scores and score components for all enrolled and imaged patients. NSTI = necrotizing soft-tissue infection.

Patient Number	Diagnosis	LRINEC Score	CRP Level (mg/L)	WBC Count (cells/µL)	Hemoglobin Level (g/dL)	Sodium Level (mmol/L)	Creatinine Level (mg/dL)	Glucose Level (mg/dL)
01	Cellulitis	9	189.6	12.8	10.5	128	1.56	217
02	NSTI	5	49.9	12.7	9.2	131	1.48	671
03	*Consent withdrawn*							
04	NSTI	6	259.4	8.2	14.3	133	2.68	57
05	Cellulitis	1*	Not taken	18.4	15.2	136	1.45	97
06	Cellulitis	6	214.9	7.7	14.4	134	0.94	117
07	NSTI	8	264.6	25.0	9.4	138	0.89	193
08	Gangrene†	5	76.0	15.3	13.0	132	1.07	315
09	Cellulitis	8	188.5	17.6	14.3	132	0.70	415
10	Cellulitis	9	>300	25.3	12.1	128	0.89	138
11	Cellulitis	2	149.6	15.1	11.2	136	2.90	124
12	NSTI	7	287	9.4	14.6	131	0.66	216
13	Osteomyelitis	8	>300	29.6	14.0	130	0.73	95
14	NSTI	2*	Not taken	18.3	11.0	136	0.83	105
15	*Consent withdrawn*							
16	NSTI	7	205.6	22.8	10.7	142	1.70	113

*Sterile, diabetes mellitus-associated gangrene.

†C-Reactive Protein (CRP) test not taken for these patients. Thus, LRINEC score calculated without C-Reactive Protein (CRP) contribution.

Supplemental Table 4. Liver disease status for all enrolled and imaged patients.

Patient Number	Diagnosis	Liver Disease
01	Cellulitis	None
02	NSTI	Fatty liver, hepatocellular carcinoma
03	*Consent withdrawn*	
04	NSTI	None
05	Cellulitis	None
06	Cellulitis	None
07	NSTI	None
08	Gangrene*	None
09	Cellulitis	None
10	Cellulitis	Hepatitis C without lesions
11	Cellulitis	None
12	NSTI	None
13	Osteomyelitis	None
14	NSTI	None
15	*Consent withdrawn*	
16	NSTI	None

*Sterile, diabetes mellitus-associated gangrene.

Supplemental Table 5. Conventional medical imaging performed as a component of standard-of-care workup for a necrotizing soft-tissue infection (NSTI). CT = computed tomography. MRI = magnetic resonance imaging.

Patient Number	Diagnosis	Imaging Performed	Findings	Diagnosis Based on Standard-of-Care Imaging Alone
01	Cellulitis	CT	Extensive edema, no loculated collections to suggest an abscess.	Cellulitis
02	NSTI	X-ray	Diffuse gas tracking along fascial planes.	None
03	*Consent withdrawn*			
04	NSTI	None		None
05	Cellulitis	CT	Extensive, diffuse thickening and subcutaneous fat stranding. No readable, rim-enhancing fluid collection to suggest abscess. No soft-tissue gas.	None
06	Cellulitis	CT	Moderate to severe subcutaneous indurative changes with edema. A small focus of subacute air within more focal soft tissue.	None
07	NSTI	CT	Skin thickening and subcutaneous stranding. No drainable abscess or soft-tissue gas.	Findings consistent with soft-tissue infection and cellulitis
08	Gangrene*	CT	Diffuse, soft-tissue edema with foci of gas.	Concerning for necrotizing infection
09	Cellulitis	CT	Subcutaneous edema. No deep fascial edema or soft-tissue air.	Findings not consistent with NSTI
10	Cellulitis	CT	Fascial thickening and perifascial fluid.	Findings concerning for NSTI
11	Cellulitis	CT	Inflammation present, multiple foci of air tracking throughout infected area.	NSTI cannot be ruled out
12	NSTI	CT, MRI	CT: Soft-tissue gas and edema noted; MRI requested for further workup. MRI: Subcutaneous air and deep fascial edema.	NSTI
13	Osteomyelitis	X-ray	Soft-tissue gas. Demineralized bone adjacent to infection site.	Concerning for Osteomyelitis
14	NSTI	CT	Cutaneous breakdown.	NSTI (Fournier's gangrene)
15	*Consent withdrawn*			
16	NSTI	CT	Extensive edema, skin thickening, and soft-tissue gas.	Neither NSTI nor osteomyelitis can be ruled out

*Sterile, diabetes mellitus-associated gangrene.

Supplemental Table 6. Causative pathogens confirmed by tissue biopsy and culture for all necrotizing soft-tissue infection (NSTI) cases.

Patient Number	Diagnosis	Tissue Culture Pathogen(s)
01	Cellulitis	
02	NSTI	*Klebsiella oxytoca*
03	*Consent withdrawn*	
04	NSTI	Beta-Hemolytic *Streptococcus*, Group C or G
05	Cellulitis	
06	Cellulitis	
07	NSTI	*Staphylococcus Aureus*; Beta-Hemolytic *Streptococcus*, Group B; *Escherichia coli*
08	Gangrene*	
09	Cellulitis	
10	Cellulitis	
11	Cellulitis	
12	NSTI	Beta-Hemolytic *Streptococcus*, Group B; *Streptococcus Milleri*, Anginosis Group; *Staphylococcus Aureus*
13	Osteomyelitis	
14	NSTI	*Enterococcus Faecalis*; *Actinotignum Schaalii*
15	*Consent withdrawn*	
16	NSTI	*Bacteroides fragilis*

*Sterile, diabetes mellitus-associated gangrene.

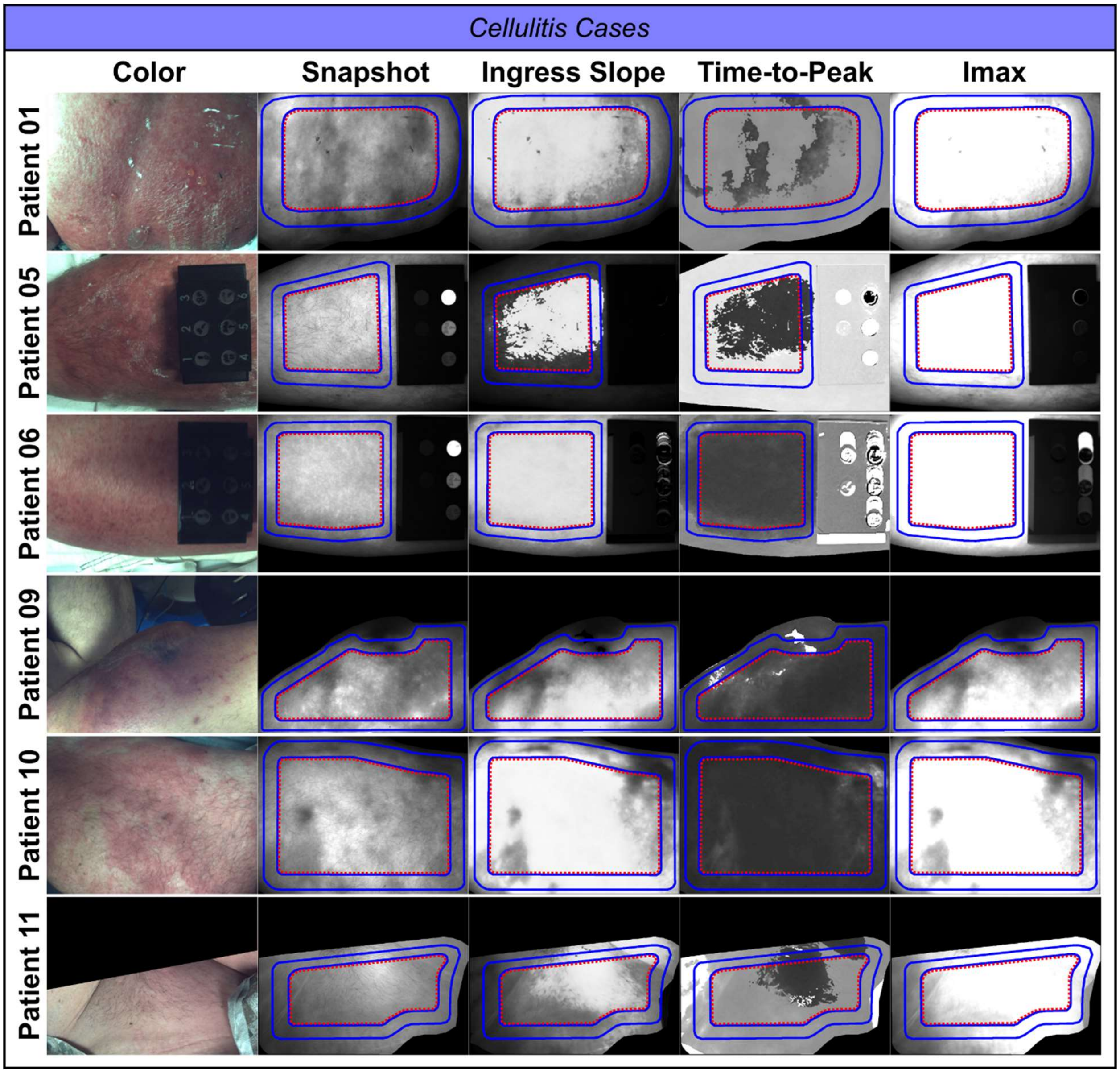

Supplemental Figure 2. White light color images (leftmost column) and corresponding ICG fluorescence parameter maps for confirmed cases of cellulitis. Regions of interest used for quantitative analysis are delineated by solid blue ("background tissue") and dashed red ("lesion") lines.

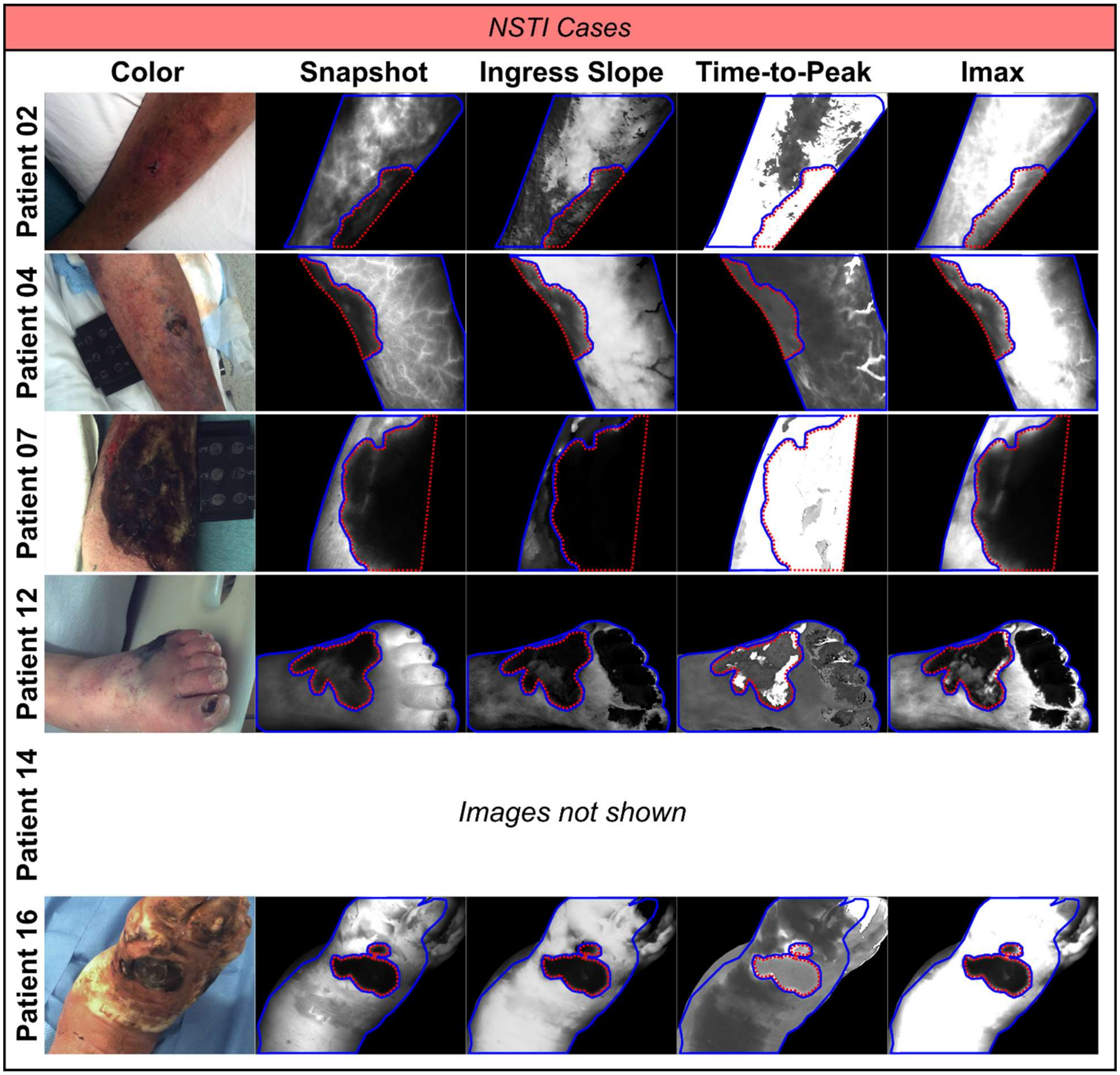

Supplemental Figure 3. White light color images (leftmost column) and corresponding ICG fluorescence parameter maps for confirmed cases of necrotizing soft-tissue infection (NSTI). Regions of interest used for quantitative analysis are delineated by solid blue ("background tissue") and dashed red ("lesion") lines.

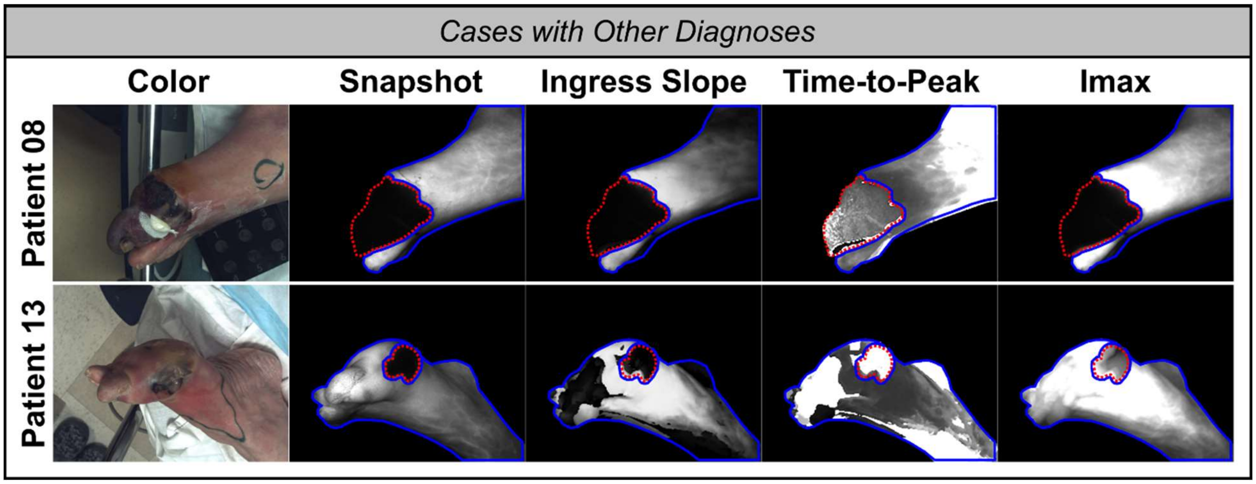

Supplemental Figure 4. White light color images (leftmost column) and corresponding ICG fluorescence parameter maps for one case of diabetes mellitus-associated gangrene (Patient 08) and one case of osteomyelitis (Patient 13). Regions of interest used for quantitative analysis are delineated by solid blue ("background tissue") and dashed red ("lesion") lines.

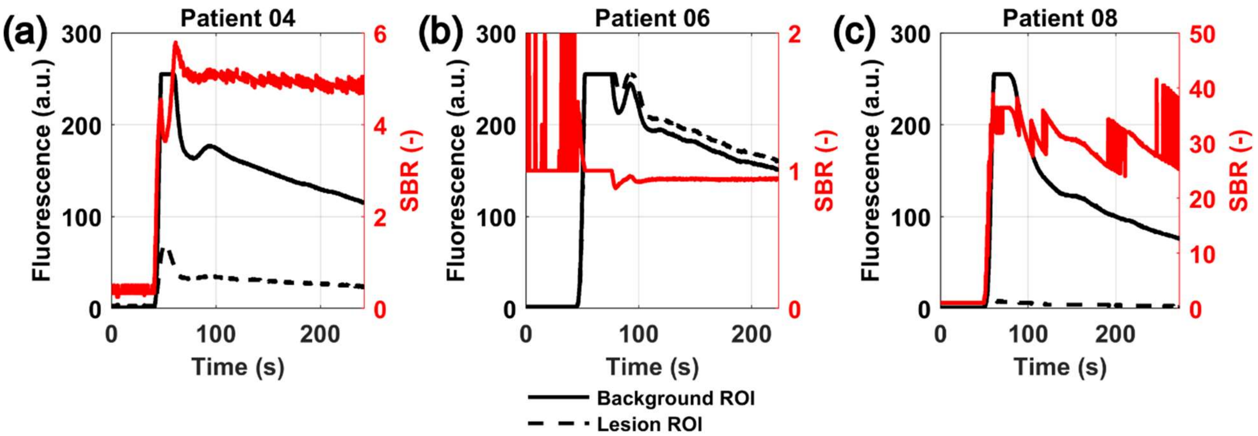

Supplemental Figure 5. ICG fluorescence intensity time profiles for representative cases of (a) necrotizing soft-tissue infection, (b) cellulitis, and (c) sterile, diabetes mellitus-associated gangrene. Regions of interest (ROIs) used to define background and lesion tissue curves are those depicted in Supplemental Figures 2-4 above. Signal-to-background ratios stabilize within approximately 60 seconds of ICG ingress into tissue vasculature.

Supplemental Table 7. Region of interest (ROI) median pixel values extracted from the "lesion" ROI in each of the four ICG fluorescence parameter maps: Snapshot, ingress slope (IS), time-to-peak intensity (TTP), and maximum intensity (IMAX). Values were used in subsequent statistical comparisons and receiver operator characteristic (ROC) curve analysis.

Patient Number	Diagnosis	ROI Median Pixel Value - Snapshot	ROI Median Pixel Value - IS	ROI Median Pixel Value - TTP	ROI Median Pixel Value - IMAX
01	Cellulitis	123.0	30.7	16.4	255.0
02	NSTI	29.0	6.6	28.8	119.4
03	*Consent withdrawn*				
04	NSTI	41.0	10.5	11.0	81.9
05	Cellulitis	192.0	35.9	9.6	255.0
06	Cellulitis	193.0	34.9	10.2	255.0
07	NSTI	14.0	0.1	100.8	14.5
08	Gangrene*	7.0	0.8	14.3	7.7
09	Cellulitis	142.0	27.9	9.5	199.3
10	Cellulitis	163.0	38.1	9.0	255.0
11	Cellulitis	138.0	26.2	16.6	255.0
12	NSTI	32.0	0.3	4.9	1.8
13	Osteomyelitis	11.0	1.3	70.3	132.6
14	NSTI	18.0	4.7	45.8	178.8
15	*Consent withdrawn*				
16	NSTI	14.0	2.9	15.8	31.2

*Sterile, diabetes mellitus-associated gangrene.
